# A Study of Frontier and Semifrontier in Intuitionistic Fuzzy Topological Spaces

**DOI:** 10.1155/2014/674171

**Published:** 2014-02-11

**Authors:** Athar Kharal

**Affiliations:** National University of Sciences and Technology (NUST), H-12, Islamabad 44000, Pakistan

## Abstract

Notions of frontier and semifrontier in intuitionistic fuzzy topology have been studied and several of their properties, characterizations, and examples established. Many counter-examples have been presented to point divergences between the IF topology and its classical form. The paper also presents an open problem and one of its weaker forms.

## 1. Introduction

The intuitionistic fuzzy sets (IFSs) were introduced by Atanassov [1] as a generalization of fuzzy sets of Zadeh [2], where besides the degree of membership *μ*_*A*_(*x*) ∈ [0,1] of each element *x* ∈ *X* to a set *A*, the degree of nonmembership *γ*_*A*_(*x*) ∈ [0,1] was also considered. IFS is a sufficiently generalized notion to include both fuzzy sets and vague sets. Fuzzy sets are IFSs but the converse is not necessarily true [1], whereas the notion of vague set defined by Gau and Buehrer [3] was proven by Bustince and Burillo [4] to be the same as IFS. IFSs have been found to be very useful in diverse applied areas of science and technology. In fact, there are situations where IFS theory is more appropriate to deal with [5]. IFSs have been applied to logic programming [6, 7], medical diagnosis [8], decision making problems [9], microelectronic fault analysis [10], and many other areas.

Tang [11] has used fuzzy topology for studying land cover changes in China. Considering the inherent nature of Geographic Information Science (GIS) phenomena, it seems more suitable to study the problem of land cover changes using intuitionistic fuzzy topology. Tang has made a heavy use of the notion of fuzzy boundary. Thus, for recasting the GIS problem in terms of Intuitionistic Fuzzy Topology makes the study of intuitionistic fuzzy frontier imperative.

In this work we study the notion of frontier in IF topology and establish several of its properties, thus providing sufficient material for researchers to utilize these concepts fruitfully. The study of weaker forms of different notions of intuitionistic Fuzzy Topology is currently underway [12–14]. Using the notion of intutionistic fuzzy semisets, we also define the notion of intuitionistic fuzzy semifrontier and characterize intuitionistic fuzzy semicontinuous functions in terms of intuitionistic fuzzy semifrontier. We extend this study further in the last section and give many properties, characterizations, and examples pertaining to the generalized notion. It is noteworthy that all the counter examples given herein are constructed upon the intuitionistic fuzzy topological space defined by Çoker [15]. In a developing field like IFS, it is interesting how the new theory differs from the old one. We have furnished two divergences from classical topology in Examples 17 and 49. An open problem and its semiversion are reported in Remarks 23 and 55.

## 2. Preliminaries

**Definition 1 (see [16])**

Let *X* be a nonempty fixed set. An intuitionistic fuzzy set (briefly IFS) *A* is an object of the form *A* = {〈*x*, *μ*(*x*), *γ*(*x*)〉 : *x* ∈ *X*}, where *μ* and *γ* are degrees of membership and nonmembership of each *x* ∈ *X*, respectively, and 0 ≤ *μ*(*x*) + *γ*(*x*) ≤ 1 for each *x* ∈ *X*. A class of all the IFS's in *X* is denoted as IFS(*X*). When there is no danger of confusion, an IFS *A* = {〈*x*, *μ*(*x*), *γ*(*x*)〉 : *x* ∈ *X*} may be written as *A* = 〈*μ*_*A*_, *γ*_*A*_〉.

**Definition 2 (see [16])**

Let *X* be a nonempty set and *A* = 〈*μ*_*A*_, *γ*_*A*_〉, *B* = 〈*μ*_*B*_, *γ*_*B*_〉 IFSs in *X*. Then

1. *A*⊆*B* if *μ*_*A*_(*x*) ≤ *μ*_*B*_(*x*) and *γ*_*A*_(*x*) ≥ *γ*_*B*_(*x*), for all *x* ∈ *X*,
2. *A* = *B* if *A*⊆*B* and *B*⊆*A*,
3. $\overset{-}{A}=\left\{\langle x,\gamma_{A}(x),\mu_{A}\left(x\right)\rangle :x\in X\right\}$,
4. *A*⋂*B* = {〈*x*, *μ*_*A*_∧*μ*_*B*_, *γ*_*A*_∨*γ*_*B*_〉 : *x* ∈ *X*} [15],
5. *A*⋃*B* = {〈*x*, *μ*_*A*_(*x*)∨*μ*_*B*_(*x*), *γ*_*A*_(*x*)∧*γ*_*B*_(*x*)〉 : *x* ∈ *X*} [15].

**Definition 3 (see [15])**

IFS's $\tilde{0}$ and $\tilde{1}$ are defined as $\tilde{0}=\left\{\langle x,0,1\rangle :x\in X\right\}$ and $\tilde{1}=\left\{\langle x,1,0\rangle :x\in X\right\}$, respectively.

**Definition 4 (see [17])**

Let *α*, *β* ∈ [0,1] and *α* + *β* ≤ 1. An intuitionistic fuzzy point (IFP for short) *x*_(*α*,*β*)_ of *X* is an IFS of *X* defined by

(1) $$\begin{array}{c} x_{\left(\alpha ,\beta \right)}\left(y\right)=\{\begin{array}{cc} \left(\alpha ,\beta \right) & \text{if}\;y=x,\\ \left(0,1\right) & \text{if}\;y\neq x. \end{array} \end{array}$$

In this case, *x* is called the support of *x*_(*α*,*β*)_ and *α* and *β* are called the *value* and the nonvalue of *x*_(*α*,*β*)_, respectively. An IFP *x*_(*α*,*β*)_ is said to belong to an IFS *A* = 〈*μ*_*A*_, *γ*_*A*_〉 in *X*, denoted by *x*_(*α*,*β*)_ ∈ *A* if *α* ≤ *μ*_*A*_(*x*) and *β* ≥ *γ*_*A*_(*x*). Clearly an intuitionistic fuzzy point can be represented by an ordered pair of fuzzy points as follows:

(2) $$\begin{array}{c} x_{\left(\alpha ,\beta \right)}=\left(x_{\alpha },1-x_{1-\beta }\right). \end{array}$$

A class of all IFP's in *X* is denoted as IFP(*X*).

**Definition 5 (see [15])**

If *B* = 〈*y*, *μ*_*B*_(*y*), *γ*_*B*_(*y*)〉 is an IFS in *Y*, then the preimage of *B* under *f*, denoted by *f* ^−1^(*B*), is the IFS in *X* defined by

(3) $$\begin{array}{c} f^{-1}\left(B\right)=\langle x,f^{-1}\left(\mu_{B}\right),f^{-1}\left(\gamma_{B}\right)\rangle . \end{array}$$

If *A* = 〈*x*, *μ*_*A*_(*x*), *γ*_*A*_(*x*)〉 is an IFS in *X*, then the image of *A* under *f*, denoted by *f*(*A*), is the IFS in *Y* defined by

(4) $$\begin{array}{c} f\left(A\right)=\langle y,f\left(\mu_{A}\right),f\left(\gamma_{A}\right)\rangle , \end{array}$$

where

(5) $$\begin{array}{c} f\left(\mu_{A}\right)\left(y\right)=\{\begin{array}{cc} \underset{x\in f^{-1}\left(y\right)}{\sup}\mu_{A}\left(x\right) & \text{if}\;f^{-1}\left(y\right)\neq \phi \\ 0 & \text{otherwise}, \end{array}\\ f\left(\gamma_{A}\right)\left(y\right)=\{\begin{array}{cc} \underset{x\in f^{-1}\left(y\right)}{\inf}\gamma_{A}\left(x\right) & \text{if}\;f^{-1}\left(y\right)\neq \phi \\ 1 & \text{otherwise}. \end{array} \end{array}$$

The concept of fuzzy topological space, first introduced by Chang in [18], was generalized to the case of intuitionistic fuzzy sets by Çoker in [15], as follows.

**Definition 6 (see [15])**

An intuitionistic fuzzy topology (IFT for short) on a nonempty set *X* is a family of IFSs in *X* satisfying the following axioms:

1. $\tilde{0},\tilde{1}\in \tau$,
2. *G*_1_⋂*G*_2_ ∈ *τ* for any *G*_1_, *G*_2_ ∈ *τ*,
3. ⋃*G*_*i*_ ∈ *τ* for any arbitrary family {*G*_*i*_ : *i* ∈ *J*}⊆*τ*.

In this case, the pair (*X*, *τ*) is called an intuitionistic fuzzy topological space (briefly, IFTS) and members of *τ* are called intuitionistic fuzzy open (briefly, IFO) sets. The complement $\overset{-}{A}$ of an IFO set *A* is called an intuitionistic fuzzy closed (IFC) set in *X*. Collection of all IFO (resp., IFC) sets in IFTS *X* is denoted as IFO(*X*) (resp., IFC(*X*)).

**Proposition 7 (see [19])**

Let *X* be an IFTS. Then the following hold:

1. $\tilde{1},\tilde{0}\in IFC\left(X\right)$,
2. *If A*_1_, *A*_2_ ∈ *IFC*(*X*)*, then A*_1_⋃*A*_2_ ∈ *IFC*(*X*),
3. *If 𝒜* ⊂ *IFC*(*X*)*, then *⋂*𝒜* ∈ *IFC*(*X*).

**Definition 8 (see [15])**

Let (*X*, *τ*) be an IFTS and *A* = 〈*μ*_*A*_, *γ*_*A*_〉 an IFS in *X*. Then the fuzzy interior and fuzzy closure of *A* are denoted and defined as

(6) $$\begin{array}{c} \text{Cl }A=⋂\left\{K:K\;\text{is}\;\text{an}\;\text{IFC}\;\text{set}\;\text{in}\;X\;\text{and}\;A\subseteq K\right\},\\ \text{Int }A=⋃\left\{G:G\;\text{is}\;\text{an}\;\text{IFO}\;\text{set}\;\text{in}\;X\;\text{and}\;G\subseteq A\right\}. \end{array}$$

**Proposition 9 (see [15])**

Let (*X*, *τ*) be an IFTS and *A*, *B* be IFSs in *X*. Then the following properties hold:

1. $\text{Int }\tilde{1}=\tilde{1}$, $\left(\text{Cl }\tilde{0}=\tilde{0}\right)$,
2. Int *A*⊆*A*, (*A*⊆Cl *A*),
3. $\bar{\text{Int }A}=\text{Cl }\overset{-}{A}$, $\left(\bar{\text{Cl }A}=\text{Int }\overset{-}{A}\right)$,
4. Int Int *A* = Int *A*, (Cl Cl *A* = Cl *A*),
5. *A*⊆*B*⇒Int *A*⊆Int *B*, (*A*⊆*B*⇒Cl *A*⊆Cl *B*),
6. Int (*A*⋂*B*) = Int *A*⋂Int *B*(Cl (*A*⋃*B*) = Cl *A*⋃Cl *B*),
7. Int (*A*⋃*B*)⊇Int *A*⋃Int *B*, (Cl (*A*⋂*B*)⊆Cl *A*⋂Cl *B*).

## 3. Intuitionistic Fuzzy Frontier

**Definition 10 (see [19])**

Let *X* be an IFTS and let *A* ∈ IFS(*X*). Then *x*_(*λ*,*μ*)_ ∈ IFP(*X*) is called an intuitionistic fuzzy frontier point (in short, IFFP) of *A* if $x_{\left(\lambda ,\mu \right)}\in \text{Cl}\;A{⋂}_{}\text{Cl}\;\overset{-}{A}$. The union of all the IFFPs of *A* is called an IF frontier of *A* and denoted by Fr *A*. It is clear that $\text{Fr}\;A=\text{Cl}\;A{⋂}_{}\text{Cl}\;\overset{-}{A}$.

**Proposition 11 (see [19])**

For each *A* ∈ *IFS*(*X*), *A*⋃Fr *A* ⊂ Cl *A*. However, the inclusion cannot be replaced by an equality.

**Theorem 12**

For an IFS *A* in an IFTS *X*, the following hold:

1. $\text{Fr }A=\text{Fr }\overset{-}{A}$,
2. If *A* is IFC then Fr *A*⊆*A*,
3. If *A* is IFO then $\text{Fr }A\subseteq \overset{-}{A}$,
4. $\bar{\text{Fr }A}=\text{Int }A{⋃}_{}\text{Int }\overset{-}{A}$.

**Proof**

(1)  $\text{Fr}\;A=\text{Cl }A{⋂}_{}\text{Cl }\overset{-}{A}=\text{Cl }\overset{-}{A}{⋂}_{}\text{Cl }A=\text{Cl }\overset{-}{A}{⋂}_{}\bar{\text{Cl}\;\overset{-}{A}}=\text{Fr }\overset{-}{A}$.

(2) $\text{Fr }A=\text{Cl }A{⋂}_{}\text{Cl}\overset{-}{\;A}\subseteq \text{Cl }A=A$; hence Fr *A*⊆*A*, if *A* is IFC set in *X*.

(3) *A* is IFO implies $\overset{-}{A}$ is IFC. By (2), $\text{Fr }\overset{-}{A}\subseteq \overset{-}{A}$ and by (1) we get $\text{Fr }A\subseteq \overset{-}{A}$.

(4) $\bar{\text{Fr }A}=\bar{\text{Cl }A{⋂}_{}\text{Cl }\overset{-}{A}}=\bar{\text{Cl }A}{⋃}_{}\bar{\text{Cl }\overset{-}{A}}=\text{Int }\overset{-}{A}⋃\text{Int }A$.

Converse of (2) and (3) of Theorem 12 is, in general, not true as is shown by the following.

**Example 13**

Let (*X*, *τ*) be the IFTS defined by Çoker (Example 3.3 [15]). We choose IFSs *A* and *B* as

(7) A={a.5,.3,b.5,.4,c.7,.1},B={a.3,.6,b.1,.5,c.2,.4}.

Then calculations give

(8) $$\begin{array}{c} \text{Fr}\;A=\left\{\frac{a}{\text{.}5,\text{.}4},\frac{b}{\text{.}4,.5},\frac{c}{\text{.}4,\text{.}2}\right\}\subseteq A, \end{array}$$

but *A* ∉ IFC(*X*). Also

(9) $$\begin{array}{c} \text{Fr}\;A=\left\{\frac{a}{\text{.}5,\text{.}4},\frac{b}{\text{.}4,\text{.}5},\frac{c}{\text{.}4,\text{.}2}\right\}\subseteq \overset{-}{A}, \end{array}$$

but *A* ∉ IFO(*X*).

**Theorem 14**

Let *A* be an IFS in an IFTS *X*. Then

1. Fr *A* = Cl *A* − Int *A*,
2. Fr Int *A*⊆Fr *A*,
3. Fr Cl *A*⊆Fr *A*,
4. Int *A*⊆*A* − Fr *A*.

**Proof**

(1)  Since $\bar{\text{Cl }\overset{-}{A}}=\text{Int }A$, therefore we have $\text{Fr }A=\text{Cl }A{⋂}_{}\text{Cl }\overset{-}{A}=\text{Cl }A-\bar{\text{Cl }\overset{-}{A}}=\text{Cl }A-\text{Int }A$. This proves (1).

(2)  $\text{Fr}\;\text{Int }A=\text{Cl Int }A{⋂}_{}\text{Cl }\bar{\left(\text{Int }A\right)}=\text{Cl Int }A{⋂}_{}\text{Cl}\;\text{Cl }\bar{A}=\text{Cl}\;\text{Int }A{⋂}_{}\text{Cl }\bar{A}\subseteq \text{Cl }A{⋂}_{}\text{Cl }\bar{A}=\text{Fr }A$.

(3) $\text{Fr}\;\text{Cl }A=\text{Cl}\;\text{Cl }A{⋂}_{}\text{Cl }\bar{\left(\text{Cl }A\right)}=\text{Cl }A{⋂}_{}\text{Cl}\;\text{Int }\bar{A}\subseteq \text{Cl }A{⋂}_{}\text{Cl }\bar{A}=\text{Fr }\bar{A}$.

(4) Consider

(10) A−Fr AA−(Cl A⋂Cl A−)=(A−Cl A)⋃(A−Cl A−)=A−Cl A−⊇Int A.

**Example 15**

To show that (2), (3), and (4) of Theorem 14 are, in general, irreversible, we choose in Example 3.3 of [15],

(11) $$\begin{array}{c} A=\left\{\frac{a}{.1,.5},\frac{b}{.8,.2},\frac{c}{.5,.3}\right\},\\ B=\left\{\frac{a}{.6,.1},\frac{b}{.5,.2},\frac{c}{.6,.3}\right\},\\ C=\left\{\frac{a}{.3,.4},\frac{b}{.3,.7},\frac{c}{.4,.4}\right\}. \end{array}$$

Then calculations give

(12) $$\begin{array}{c} \text{Fr}\;\text{Int }A=\tilde{0}\neq \tilde{1}=\text{Fr }A,\\ \text{Fr }B=\left\{\frac{a}{.2,.5},\frac{b}{.4,.5},\frac{c}{.4,.4}\right\}⊈\tilde{0}=\text{Fr}\;\text{Cl }B\\ C-\text{Fr }C=\left\{\frac{a}{.3,.5},\frac{b}{.3,.7},\frac{c}{.2,.4}\right\}⊈\tilde{0}=\text{Int }C. \end{array}$$

**Remark 16**

In general topology, the following hold:

(13) $$\begin{array}{c} \text{Fr }A⋂\text{Int }A=\phi ,\\ \text{Int }A⋃\text{Fr }A=\text{Cl }A,\\ \text{Int }A⋃\text{Int }\overset{-}{A}⋃\text{Fr }A=X. \end{array}$$

Whereas in IF topology, we give counter-examples to show that these may not hold in general.

**Example 17**

In Example 3.3 of [15], we choose

(14) $$\begin{array}{c} A=\left\{\frac{a}{.7,.2},\frac{b}{.9,.1},\frac{c}{.9,.1}\right\},\\ B=\left\{\frac{a}{.7,.3},\frac{b}{.5,.4},\frac{c}{.4,.3}\right\}, \end{array}$$

then we have

(15) Fr A⋂Int A={a.2,.5,b.3,.6,c.3,.4}≠0~,Int B⋃Int B−⋃Fr B ={a.4,.5,b.5,.4,c.2,.4}⋃0~⋃{a.5,.4,b.4,.5,c.4,.2} ={a.5,.4,b.5,.4,c.4,.2}≠1~.

We now investigate the expression Fr (*A*⋃*B*). We first show that the equality Fr (*A*⋃*B*) = Fr *A*⋃Fr *B* does not hold and is in fact an irreversible inclusion.

**Theorem 18**

Let *A* and *B* be IFSs in an IFTS *X*. Then Fr (*A*⋃*B*)⊆Fr *A*⋃Fr *B*.

**Proof**

Consider

(16) Fr (A⋃B) =Cl (A⋃B)⋂Cl (A⋃B¯) ⊆(Cl A⋃Cl B)⋂(Cl A−⋂Cl B¯) =[(Cl A⋃Cl B)⋂Cl A−]⋂[(Cl A⋃Cl B)⋂Cl B¯] =[(Cl A⋂Cl A−)⋃(Cl B⋂Cl A−)]  ⋂[(Cl A⋂Cl B¯)⋃(Cl B⋂Cl B¯)] =[Fr A⋃(Cl B⋂Cl A−)]⋂[(Cl A⋂Cl B¯)⋃Fr B] =(Fr A⋃Fr B)⋂((Cl B⋂Cl A−)⋃(Cl A⋂Cl B¯)) ⊆(Fr A⋃Fr B).

The converse of Theorem 18 is in general not true, as is shown by the following.

**Example 19**

In Example 3.3 of [15], if we choose

(17) $$\begin{array}{c} A=\left\{\frac{a}{.5,.2},\frac{b}{.5,.1},\frac{c}{.8,.2}\right\},\;\;B=\left\{\frac{a}{.6,.2},\frac{b}{.6,.1},\frac{c}{.7,.1}\right\}, \end{array}$$

then calculations give

(18) Fr A⋃Fr B={a.2,.5,b.4,.5,c.4,.4}⋃{a.2,.5,b.3,.6,c.3,.4}={a.2,.5,b.4,.5,c.4,.4}⊈{a.2,.5,b.3,.6,c.3,.4}=Fr (A⋃B).

Again if we choose

(19) $$\begin{array}{c} A=\left\{\frac{a}{.3,.4},\frac{b}{.4,.5},\frac{c}{.2,.1}\right\},\;\;B=\left\{\frac{a}{.5,.4},\frac{b}{.2,.2},\frac{c}{.5,.5}\right\}, \end{array}$$

then

(20) Fr A⋂Fr B=1~⋂1~=1~⊈{a.5,.4,b.4,.5,c.4,.2}=Fr ({a.3,.4,b.2,.5,c.2,.5})=Fr (A⋂B),

and choosing

(21) $$\begin{array}{c} A=\left\{\frac{a}{.1,.7},\frac{b}{.8,.1},\frac{c}{.2,.8}\right\},\;\;B=\left\{\frac{a}{.5,.4},\frac{b}{.5,.1},\frac{c}{.9,.1}\right\}, \end{array}$$

we get

(22) Fr (A⋂B)=Fr ({a.1,.7,b.5,.1,c.2,.8})=1~⊈{a.5,.4,b.4,.5,c.4,.2}=1~⋂{a.5,.4,b.4,.5,c.4,.2}=Fr A⋂Fr B.

However, we have the following.

**Theorem 20**

For any IFSs *A* and *B* in an IFTS *X*,

(23) $$\begin{array}{c} \text{Fr }\left(A⋂B\right)\subseteq \left(\text{Fr }A⋂\text{Cl}\;B\right)⋃\left(\text{Fr }B⋂\text{Cl }A\right). \end{array}$$

**Proof**

Consider

(24) Fr (A⋂B) =Cl (A⋂B)⋂Cl (A⋂B¯) ⊆(Cl A⋂Cl B)⋂(Cl A−⋃Cl B¯) =[(Cl A⋂Cl B)⋂Cl A−]⋃[(Cl A⋂Cl B)⋂Cl B¯] =(Fr A⋂Cl B)⋃(Fr B⋂Cl A).

**Example 21**

To show that the converse of Theorem 20 is in general not true, in Example 3.3 of [15] we choose

(25) $$\begin{array}{c} A=\left\{\frac{a}{.9,.1},\frac{b}{.3,.6},\frac{c}{.2,.6}\right\},\;\;B=\left\{\frac{a}{.3,.4},\frac{b}{.7,.2},\frac{c}{.2,.6}\right\}, \end{array}$$

then calculations give

(26) (Fr A⋂Cl B)⋃(Fr B⋂Cl A) =(1~⋂1~)⋃(1~⋂1~) =1~⊈{a.5,.4,b.3,.6,c.3,.2}=Fr (A⋂B).

**Theorem 22**

For any IFS *A* in an IFTS *X*,

1. Fr Fr *A*⊆Fr *A*,
2. Fr Fr Fr *A*⊆Fr Fr *A*.

**Remark 23**

We checked (2) of Theorem 22 on a large number of IFTSs, no counter-example could be found to establish the irreversibility of inequality. Therefore, it is conjectured that the equality in (2) holds and its proof is sought. However, the converse of (1) is, in general, not true as is shown by the following.

**Example 24**

In Example 3.3 of [15], if we choose

(27) $$\begin{array}{c} A=\left\{\frac{a}{.3,.2},\frac{b}{.4,.6},\frac{c}{.6,.1}\right\}, \end{array}$$

then, we have

(28) $$\begin{array}{c} \text{Fr }A=\tilde{1}⊈\tilde{0}=\text{Fr}\;\text{Fr }A. \end{array}$$

**Theorem 25 (see [13])**

Let *X* and *Y* be product related IFTSs. Then, for an IFS *A* of *X* and an IFS *B* of *Y*,

1. Cl (*A* × *B*) = Cl *A* × Cl *B*,
2. Int (*A* × *B*) = Int *A* × Int *B*.

**Theorem 26**

Let *X*_*i*_, *i* = 1,2,…, *n* be a family of product related IFTSs. If *A*_*n*_⊆*X*_*n*_, then

(29) Fr ∏i=1nAi=[Fr A1×Cl A2×⋯×Cl An]    ⋃[Cl A1×Fr A2×Cl A3×⋯×Cl An]⋃⋯    ⋃[Cl A1×Cl A2×⋯×Fr An].

**Proof**

We use Theorem 14  (1) and Theorem 25 to prove this.

It suffices to prove this for *n* = 2. Consider

(30) Fr (A1×A2) =Cl (A1×A2)−Int (A1×A2) =(Cl A1×Cl A2)−(Int A1×Int A2) =(Cl A1×Cl A2)−(Int A1⋂Cl A1×Int A2⋂Cl A2) =(Cl A1×Cl A2)−[(Int A1×Cl A2)⋂(Cl A1×Int A2)] =[(Cl A1×Cl A2)−Int A1×Cl A2]  ⋃[(Cl A1×Cl A2)−(Cl A1×Int A2)] =[(Cl A1−Int A2)×Cl A2]⋃[Cl A1×(Cl A2−Int A2)] =(Fr A1×Cl A2)⋃(Cl A1×Fr A2).

**Definition 27 (see [19])**

Let (*X*, *τ*) be an IFTS, *A* ∈ IFS(*X*) and let *x*_(*λ*,*μ*)_ ∈ IFP(*X*). Then *A* is called an intuitionistic Q-neighborhood (in short, IQN) of *x*_(*λ*,*μ*)_ if there is a *B* ∈ *τ* such that *x*_(*λ*,*μ*)_ *qB* ⊂ *A*. The family of all the IQNs of *x*_(*λ*,*μ*)_ is called the system of IQNs of *x*_(*λ*,*μ*)_ and denoted by *𝒩*_IQ_(*x*_(*λ*,*μ*)_).

**Definition 28 (see [19])**

Let *X* be an IFTS and let *A* ∈ IFS(*X*). Then *x*_(*λ*,*μ*)_ ∈ IFP(*X*) is called an intuitionistic fuzzy adherence point (in short, IFAP) of *A* if for each *U* ∈ *𝒩*_IQ_(*x*_(*λ*,*μ*)_), *UqA*.

**Definition 29 (see [19])**

Let *X* be an IFTS and *A* ∈ IFS(*X*). Then *x*_(*λ*,*μ*)_ ∈ IFP(*X*) is called an intuitionistic fuzzy accumulation point of *A* if it satisfies the following conditions:

1. *x*_(*λ*,*μ*)_ is an IFAP of *A*,
2. if *x*_(*λ*,*μ*)_ ∈ *A*, then for each *U* ∈ *𝒩*_IQ_(*x*_(*λ*,*μ*)_), *U* and *A* are quasicoincident at some point *y* ∈ *X* such that *y* ≠ *x*.

The union of all the intuitionistic fuzzy accumulation points of *A* is called the derived set of *A* and is denoted by *A* ^*d*^. It is clear that *A* ^*d*^ ⊂ Cl *A*.

**Proposition 30 (see [19])**

For any IFS *A* in an IFTS *X*, Cl *A* = *A* ⋃ *A* ^*d*^.

**Corollary 31 (see [19])**

Let *A* ∈ *IFS*(*X*). Then *A* ∈ *IFC*(*X*) iff *A* ^*d*^ ⊂ *A*.

**Definition 32 (see [15])**

Let (*X*, *τ*) and (*Y*, *ϕ*) be two IFTSs and *f* : *X* → *Y*, a function. Then *f* is said to be intuitionistic fuzzy continuous if the preimage of each IFS in *ϕ* is in *τ*.

**Theorem 33**

Let *f* : *X* → *Y* be a mapping. Then the following are equivalent:

1. *f* : *X* → *Y* is IF continuous,
2. *f*(*A* ^*d*^)⊆Cl *f*(*A*), for any IFS *A* in *X*.

**Proof**

(1)⇒(2) Suppose that *f* is IF continuous. Let *A* be an IFS in *X*. Since Cl *f*(*A*) is IF closed in *Y*, *f* ^−1^(Cl *f*(*A*)) is IF closed in *X*. *A*⊆*f* ^−1^(Cl *f*(*A*)) gives Cl *A*⊆Cl *f* ^−1^(Cl *f*(*A*)) = *f* ^−1^(Cl *f*(*A*)). Therefore, *f*(*A* ^*d*^)⊆*f*(Cl *A*)⊆*ff* ^−1^(Cl *f*(*A*))⊆Cl *f*(*A*). Consequently, *f*(*A* ^*d*^)⊆Cl *f*(*A*).

(2)⇒(1) Suppose *f*(*A* ^*d*^)⊆Cl *f*(*A*), where *A* is an IFS in *X*. Let *B* be any IF closed set in *Y*. We show that *f* ^−1^(*B*) is IF closed in *X*. By our hypothesis, *f*([*f* ^−1^(*B*)]^*d*^)⊆Cl *f*(*f* ^−1^(*B*))⊆Cl *B* = *B* or *f*([*f* ^−1^(*B*)]^*d*^)⊆*B* gives [*f* ^−1^(*B*)]^*d*^⊆*f* ^−1^(*f*[*f* ^−1^(*B*)]^*d*^)⊆*f* ^−1^(*B*) or [*f* ^−1^(*B*)]^*d*^⊆*f* ^−1^(*B*) implies *f* ^−1^(*B*) is IF closed in *X*. Thus, *f* is IF continuous.

**Theorem 34**

Let *f* : *X* → *Y* be an IF continuous mapping. Then Fr *f* ^−1^(*B*)⊆*f* ^−1^(Fr *B*), for any IFS *B* in *Y*.

**Proof**

Suppose that *f* is IF continuous. Let *B* be an IFS in *Y*. Then

(31) Fr f−1(B)=Cl f−1(B)⋂Cl f−1(B)¯⊆Cl f−1(Cl B)⋂Cl f−1(B¯)⊆Cl f−1(Cl B)⋂Cl f−1(Cl B¯)=f−1(Cl B)⋂f−1(Cl B¯)=f−1(Cl B⋂Cl B¯)=f−1(Fr B).

Therefore Fr *f* ^−1^(*B*)⊆*f* ^−1^(Fr *B*).

**Lemma 35**

Let *A*⊆*B* and *B* ∈ *IFC* *S*(*X*). Then Fr *A*⊆*B*.

**Proof**

Since *A*⊆*B* implies Cl *A*⊆Cl *B*, we have $\text{Fr }A=\text{Cl }A{⋂}_{}\text{Cl }\overset{-}{A}\subseteq \text{Cl }B{⋂}_{}\text{Cl }\bar{A}\subseteq \text{Cl }B=B$.

**Definition 36 (see [15])**

Let (*X*, *τ*) and (*Y*, *ϕ*) be two IFTSs and *f* : *X* → *Y*, a function. Then *f* is said to be fuzzy open if the image of each IFS in *τ* is in *ϕ*.

**Theorem 37**

Let *f* : *X* → *Y* be an IFO mapping and *B* an IFS in *Y*. Then *f* ^−1^(Fr *B*)⊆Fr *f* ^−1^(*B*).

**Proof**

Suppose *f* is IFO and *B* is an IFS in *Y*. Put

(32) $$\begin{array}{c} A=\bar{\text{Fr }f^{-1}\left(B\right)}. \end{array}$$

Then *A* is IF open and therefore *f*(*A*) is IF open in *Y*. This gives $\bar{f\left(A\right)}\in \text{IFCS}(Y)$. From (32), we get $B\subseteq \bar{f\left(A\right)}$. Then by Lemma 35, we have

(33) f−1(Fr B)⊆f−1(f(A)¯)⊆A¯=Fr f−1(B)¯¯=Fr f−1(B).

Consequently, we have *f* ^−1^(Fr *B*)⊆Fr *f* ^−1^(*B*).

## 4. Intuitionistic Fuzzy Semifrontier

Levine [20] generalized the notion of open sets as semiopen sets. His impetus for the generalization was to develop a wider framework for the study of continuity and its different variants. Interestingly, his work also found application in the field of digital topology [21], though it was never in sight at the time of inception of semitype notions (technically known as weaker notions). For example, it was found that digital line is a *T*_1/2_-space [22], which is a weaker separation axiom based upon semiopen sets. Fuzzy digital topology [23] was introduced by Rosenfeld, which demonstrated the need for the fuzzification of weaker forms of notions of classical topology. Azad [24] carried out this fuzzification in 1981, and thus initiated the study of the concepts of fuzzy semiopen and fuzzy semiclosed sets. Intutionistic Fuzzy Topology, being a relatively new field also followed the trajectory of its nearest analogue: fuzzy topology. Thus study of weaker forms of different notions in the settings of Intuitionistic Fuzzy Topology is currently a very active area of research [13, 14]. In this section, we generalize the definitions and results of intuitionistic fuzzy frontier in the intuitionistic fuzzy semisettings.

**Definition 38 (see [12])**

An IFS *A* in an IFTS (*X*, *τ*) is called an intuitionistic fuzzy semiopen set (IFSOS) if

(34) $$\begin{array}{c} A\subseteq \text{Cl}\;\text{Int }\left(A\right). \end{array}$$

An IFS *A* is called an intuitionistic fuzzy semiclosed set if the complement of *A* is an IFSOS.

**Definition 39**

The semiclosure and semi-interior of an IFS *A* in an IFTS (*X*, *τ*) are denoted and defined as

(35) $$\begin{array}{c} s\text{Cl }A=⋂\left\{B\;|\;A\subseteq B,B\;\text{is}\;\text{IFSC}\;\text{set}\right\},\\ s\text{Int }A=⋃\left\{C\;|\;C\subseteq A,C\;\text{is}\;\text{IFSO}\;\text{set}\right\}. \end{array}$$

**Theorem 40**

For an IFS *A* in IFTS *X*, the following hold:

1. *s*Cl *A* = *A*⋃Int Cl *A*,
2. *s*Int *A* = *A*⋂Cl Int *A*.

**Proof**

(1)  Let *A* be an IFS in *X*. From Int (Cl (*A*⋃Int Cl *A*)) = Int (Cl *A*⋃Cl Int Cl *A*) = Int Cl *A*⊆*A*⋃Int Cl *A*, it follows that *A*⋃Int Cl *A* is an IFSC set. Hence, *s*Cl *A*⊆*A*⋃Int Cl *A*. Since *s*Cl *A* is IFSC, we have Int Cl *A*⊆Int Cl *s*Cl *A*⊆*s*Cl *A*. Thus *A*⋃Int Cl *A*⊆*s*Cl *A*.

(2) This can be proved in a similar manner as (1).

**Definition 41**

Let *A* be an IFS in IFTS *X*. Then the intuitionistic fuzzy semifrontier of *A* is defined as $s\text{Fr }A=s\text{Cl }A{⋂}_{}s\text{Cl }\overset{-}{A}$. Obviously, *s*Fr *A* is an IFSC set.

**Remark 42**

In the following theorems, we note that almost all the properties related to intuitionistic fuzzy semi-interior, intuitionistic fuzzy semi-closure and intuitionistic fuzzy semifrontier are analogous to their counterparts in Intuitionistic Fuzzy Topology, and hence proofs of most of them are not given.

**Theorem 43**

For IFSs *A* and *B* in an IFTS *X*, one has

1. $\bar{s\text{Int }A}=s\text{Cl }\overset{-}{A}$,
2. $\bar{s\text{Cl }A}=s\text{Int }\overset{-}{A}$,
3. *s*Cl  *s*Cl *A* = *s*Cl *A*,
4. *s*Int  *s*Int *A* = *s*Int *A*,
5. *s*Int (*A*⋃*B*)⊇*s*Int *A*⋃*s*Int *B*,
6. *s*Int (*A*⋂*B*) = *s*Int *A*⋂*s*Int *B*,
7. *s*Cl (*A*⋃*B*) = *s*Cl *A*⋃*s*Cl *B*,
8. *s*Cl (*A*⋂*B*)⊆*s*Cl *A*⋂*s*Cl *B*.

**Proof**

(5) *s*Int *A* and *s*Int *B* are both IFSO sets and *A*⊆*A*⋃*B*, *B*⊆*A*⋃*B* implies *s*Int *A*⊆*s*Int (*A*⋃*B*) and *s*Int *B*⊆*s*Int (*A* ⋃ *B*). In Combination, *s*Int *A*⋃*s*Int *B*⊆*s*Int (*A*⋃*B*) or

(36) $$\begin{array}{c} s\text{Int }\left(A⋃B\right)⊇s\text{Int }A⋃s\text{Int }B. \end{array}$$

(6) *A*⋂*B*⊆*A* and *A*⋂*B*⊆*B* imply *s*Int (*A*⋂*B*)⊆*s*Int *A*⋂*s*Int *B*. Conversely, *s*Int *A*⊆*A* and *s*Int *B*⊆*B* imply *s*Int *A*⋂*s*Int *B*⊆*A*⋂*B* and *s*Int *A*⋂*s*Int *B* is IFSO. But *s*Int (*A*⋂*B*) is the largest IFSO set contained in *A*⋂*B*; hence *s*Int *A*⋂*s*Int *B*⊆*s*Int (*A*⋂*B*). This gives the equality.

(7) This follows easily from (2).

(8) Since *A*⋂*B*⊆*A*, *A*⋂*B*⊆*B*

(37) ⟹sCl (A⋂B)⊆sCl A,sCl (A⋂B)⊆sCl B⟹sCl (A⋂B)⊆sCl A⋂sCl B.

In the following theorem, (1)–(5) are analogues of Theorem 12, and hence we omit their proofs.

**Theorem 44**

For an IFS *A* in IFTS *X*, the following hold:

1. $s\text{Fr }A=s\text{Fr}\;\overset{-}{A}$,
2. if *A* is IFSC, then *s*Fr *A*⊆*A*,
3. if *A* is IFSO, then $s\text{Fr }A\subseteq \overset{-}{A}$,
4. let *A*⊆*B* and *B* ∈ *IFS* *C*(*X*) (resp., *B* ∈ *IFS* *O*(*X*)). Then *s*Fr *A*⊆*B* (resp., $s\text{Fr }A\subseteq \overset{-}{B}$), where *IFS* *C*(*X*) (resp., *IFS* *O*(*X*)) denotes the class of intuitionistic fuzzy semi-closed (resp. intuitionistic fuzzy semiopen) sets in *X*,
5. $\bar{s\text{Fr }A}=s\text{Int }A{⋃}_{}s\text{Int }\overset{-}{A}$,
6. *s*Fr *A*⊆Fr *A*,
7. *s*Cl *s*Fr *A*⊆Fr *A*.

**Proof**

(6) Since *s*Cl *A*⊆Cl *A* and $s\text{Cl }\overset{-}{A}\subseteq \text{Cl }\overset{-}{A}$, then we have

(38) $$\begin{array}{c} s\text{Fr }A=s\text{Cl }A⋂s\text{Cl }\overset{-}{A}\subseteq \text{Cl }A⋂\text{Cl }\overset{-}{A}=\text{Fr }A. \end{array}$$

(7) $s\text{Cl}\;s\text{Fr }A=s\text{Cl }\left(s\text{Cl }A{⋂}_{}s\text{Cl }\overset{-}{A}\right)\subseteq s\text{Cl}\;s\text{Cl }A{⋂}_{}s\text{Cl }s\text{Cl }\overset{-}{A}=s\text{Cl }A{⋂}_{}s\text{Cl }\overset{-}{A}=s\text{Fr }A\subseteq \text{Fr }A$.

The converse of (2), (3), (6), and (7) of Theorem 44 is, in general, not true as is shown by the following.

**Example 45**

In Example 3.3 of [15], we choose

(39) $$\begin{array}{c} A=\left\{\frac{a}{.4,.4},\frac{b}{.8,.2},\frac{c}{.9,.1}\right\},\;\;B=\left\{\frac{a}{.2,.8},\frac{b}{.2,.7},\frac{c}{.1,.2}\right\},\\ C=\left\{\frac{a}{.8,.2},\frac{b}{.6,.1},\frac{c}{.4,.2}\right\},\;\;D=\left\{\frac{a}{.9,.1},\frac{b}{.8,.2},\frac{c}{.4,.3}\right\}, \end{array}$$

then calculations give

(40) sFr A=A−⊆A  but  A∉IFSC(X),sFr B=B⊆B−  but  B∉IFSO(X),Fr C={a.2,.5,b.3,.6,c.3,.4}⊈C−=sFr C,Fr D={a.2,.5,b.3,.6,c.3,.4}⊈{a.1,.9,b.2,.8,c.3,.4}=sCl ({a.1,.9,b.2,.8,c.3,.4})=sCl sFr D.

The following is an analogue of Theorem 14.

**Theorem 46**

Let *A* be an IFS in IFTS *X*. Then one has

1. *s*Fr *A* = *s*Cl *A* − *s*Int *A*,
2. *s*Fr *s*Int *A*⊆*s*Fr *A*,
3. *s*Fr *s*Cl *A*⊆*s*Fr *A*,
4. *s*Int *A*⊆*A* − *s*Fr *A*.

**Example 47**

To show that (2), (3), and (4) of Theorem 46, are, in general, irreversible, in Example 3.3 of [15], we choose

(41) $$\begin{array}{c} A=\left\{\frac{a}{.9,.1},\frac{b}{.4,.1},\frac{c}{.3,.1}\right\},\;\;B=\left\{\frac{a}{.3,.4},\frac{b}{.3,.6},\frac{c}{.5,.4}\right\},\\ C=\left\{\frac{a}{.3,.5},\frac{b}{.3,.7},\frac{c}{.1,.3}\right\}, \end{array}$$

then the calculations show

(42) sFr A=1~⊈0~=sFr (0~)=sFr sInt A,sFr B=1~⊈0~=sFr 0~=sFr sCl B,C−sFr C=C−C=C⊈0~=sInt C.

**Remark 48**

In general topology, the following hold:

(43) $$\begin{array}{c} s\text{Fr }A⋂s\text{Int }A=\phi ,\\ s\text{Int }A⋃s\text{Fr }A=\text{Cl }A,\\ s\text{Int }A⋃s\text{Int}{\;A}^{c}⋃s\text{Fr }A=X. \end{array}$$

Whereas, in Intuitionistic Fuzzy Topology, we give counter-examples to show that these may not hold in general.

**Example 49**

In Example 3.3 of [15], we choose

(44) $$\begin{array}{c} A=\left\{\frac{a}{.4,.3},\frac{b}{.8,.1},\frac{c}{.9,.1}\right\},\;\;B=\left\{\frac{a}{.6,.2},\frac{b}{.7,.3},\frac{c}{.8,.1}\right\},\\ C=\left\{\frac{a}{.6,.3},\frac{b}{.7,.2},\frac{c}{.3,.4}\right\}, \end{array}$$

then calculations give

(45) sFr A⋂sInt A=A−⋂A=A−≠0~,sInt B⋃sFr B=B⋃B−=B≠1~=sCI B,sInt C⋃sInt C−⋃sFr C=C⋃0~⋃C−={a.6,.3,b.7,.2,c.4,.3}≠1~.

**Theorem 50**

Let *A* and *B* be IFSs in an IFTS *X*. Then *s*Fr (*A*⋃*B*)⊆*s*Fr *A*⋃*s*Fr *B*.

The converse of Theorem 50 is, in general, not true as is shown by the following.

**Example 51**

In Example 3.3 of [15], we choose

(46) $$\begin{array}{c} A=\left\{\frac{a}{.1,.5},\frac{b}{.2,.8},\frac{c}{.3,.4}\right\},\;\;B=\left\{\frac{a}{.6,.3},\frac{b}{.5,.4},\frac{c}{.7,.1}\right\}, \end{array}$$

then calculations show

(47) sFr A⋃sFr B=A⋃B−={a.3,.5,b.4,.5,c.3,.4}⊈B−=sFr B=sFr (A⋃B).

However, we have the following theorem which is an analogue of Theorem 20.

**Theorem 52**

For IFSs *A* and *B* in IFTS *X*, one has

(48) $$\begin{array}{c} s\text{Fr }\left(A⋂B\right)\subseteq \left(s\text{ Fr }A⋂s\text{ Cl }B\right)⋃\left(s\text{ Fr }B⋂s\text{ Cl }A\right). \end{array}$$

**Corollary 53**

For IFSs *A* and *B* in IFTS *X*, one has

(49) $$\begin{array}{c} s\text{ Fr }\left(A⋂B\right)\subseteq s\text{ Fr }A⋃s\text{ Fr }B. \end{array}$$

The analogue of Theorem 22 is the following theorem, the proof of which is easy to establish.

**Theorem 54**

For an IFS *A* in IFTS *X*, one has

1. *s*Fr *s*Fr *A*⊆*s*Fr *A*,
2. *s*Fr *s*Fr *s*Fr *A*⊆*s*Fr *s*Fr *A*.

**Remark 55**

As in the case of Theorem 22(2) we also do not know whether the equality in Theorem 54(2) holds or not. So we leave these as open problems. However, the converse of (1) is, in general, not true as is shown by the following.

**Example 56**

In Example 3.3 of [15], we choose

(50) $$\begin{array}{c} A=\left\{\frac{a}{.1,.3},\frac{b}{.8,.2},\frac{c}{.1,.7}\right\}, \end{array}$$

then it is easy to see that $s\text{Fr }A=\tilde{1}⊈\tilde{0}=s\text{Fr }s\text{Fr }A$.

**Definition 57**

An IFS *A* in IFTS *X* is called an intuitionistic fuzzy semi-Q-neighborhood of an IFP *e* if there exists an IFSO set *B* in *X*, such that *e* *qB*⊆*A*.

**Theorem 58**

An IFP *e* = *x*_(*α*,*β*)_ ∈ *s*Cl *A* if each intuitionistic fuzzy semi-Q-neighborhood of *e* is quasicoincident with *A*.

**Proof**

*x*_(*α*,*β*)_ ∈ *s*Cl *A* if for every fuzzy closed set *C*⊇*A*, *x*_(*α*,*β*)_ ∈ *C*. This gives *C*(*x*_(*α*,*β*)_)⊇*A*(*x*_(*α*,*β*)_). Equivalently, *x*_(*α*,*β*)_ ∈ *s*Cl *A* if for every IFSO set $B\subseteq \overset{-}{A}$, $B\left(x_{\left(\alpha ,\beta \right)}\right)\subseteq \overset{-}{A}\left(x_{\left(\alpha ,\beta \right)}\right)$. That is, for every fuzzy open set *B* satisfying $B\left(x_{\left(\alpha ,\beta \right)}\right)⊇\overset{-}{A}$, *B* is not contained in $\overset{-}{A}$, or $Bq\bar{\overset{-}{A}}=A$. Thus, *x*_(*α*,*β*)_ ∈ *s*Cl *A* if every IFO Q-neighborhood *B* of *x*_(*α*,*β*)_ is quasi-coincident with *A*.

**Definition 59**

An IFP *e* is called a semiadherence point of an IFS *A* if every intuitionistic fuzzy semi-Q-neighborhood of *e* is quasi-coincident with *A*.

**Definition 60**

An IFP *e* is called a semiaccumulation point of an IFS *A* if *e* is a semi-adherence point of *A* and every semi-Q-neighborhood of *e* and *A* is quasi-coincident at some point different from supp(*e*), whenever *e* ∈ *A*. The union of all the semi-accumulation points of *A* is called the intuitionistic fuzzy semiderived set of *A*, denoted as *A* ^*sd*⁡^. It is evident that *A* ^*sd*⁡^⊆*s*Cl *A*.

**Proposition 61**

Let *A* be an IFS in *X*, then *s* Cl *A* = *A*⋃*A* ^*sd*^.

**Proof**

Let *Ω* = {*e* | *e*  is  a  semi-adherence  point  of  *A*}. Then from Theorem 58, *s*Cl *A* = ⋃*Ω*. On the other hand, *e* ∈ *Ω* is either *e* ∈ *A* or *e* ∉ *A*; for the latter case, by Definition 60, *e* ∈ *A* ^*sd*⁡^; hence *s*Cl *A* = ⋃*Ω* ⊂ *A*⋃*A* ^*sd*⁡^. The reverse inclusion is obvious.

**Corollary 62**

For any IFS *A* in an IFTS *X*, *A* is IFSC if *A* ^*sd*^⊆*A*.

**Definition 63**

Let *f* : *X* → *Y* be a function from an IFTS *X* to another IFTS *Y*. Then *f* is said to be an intuitionistic fuzzy semicontinuous function if *f* ^−1^(*A*) is IFSO in *X* for each IFO set *A* in *Y*.

**Theorem 64**

Let *f* : *X* → *Y* be a function. Then the following are equivalent:

1. *f* : *X* → *Y* is intuitionistic fuzzy semi-continuous,
2. *f*(*A* ^*sd*^)⊆*s*Cl *f*(*A*), for any IFS *A* in *X*.

**Proof**

(1)⇒(2) Suppose that *f* is intuitionistic fuzzy semi-continuous. Let *A* be an IFS in *X*. Since *s*Cl *f*(*A*) is IFC in *Y*, *f* ^−1^(*s*Cl *f*(*A*)) is IFSC in *X*. *A*⊆*f* ^−1^(*s*Cl *f*(*A*)) gives *s*Cl *A*⊆*s*Cl *f* ^−1^(*s*Cl *f*(*A*)) = *f* ^−1^(*s*Cl *f*(*A*)). Therefore, *f*(*A* ^*sd*⁡^)⊆*f*(*s*Cl *A*)⊆*ff* ^−1^(*s*Cl *f*(*A*))⊆*s*Cl *f*(*A*). Consequently, *f*(*A* ^*sd*⁡^)⊆*s*Cl *f*(*A*).

(2)⇒(1) Suppose *f*(*A* ^*sd*⁡^)⊆*s*Cl *f*(*A*). Letting *B* be any IFC set in *Y*, we show that *f* ^−1^(*B*) is IFSC in *X*. By our hypothesis, *f*([*f* ^−1^(*B*)]^*sd*⁡^)⊆*s*Cl *f*(*f* ^−1^(*B*))⊆*s*Cl *B* = *B* or *f*([*f* ^−1^(*B*)]^*sd*⁡^)⊆*B* gives [*f* ^−1^(*B*)]^*sd*⁡^⊆*f* ^−1^(*f*[*f* ^−1^(*B*)]^*sd*⁡^)⊆*f* ^−1^(*B*) or [*f* ^−1^(*B*)]^*sd*⁡^⊆*f* ^−1^(*B*) implies *f* ^−1^(*B*) is IFSC in *X*. Thus, *f* is intuitionistic fuzzy semi-continuous.

**Theorem 65**

Let *f* : *X* → *Y* be a intuitionistic fuzzy semi-continuous function. Then one has

(51) $$\begin{array}{c} s\text{Fr }f^{-1}\left(B\right)\subseteq f^{-1}\left(s\text{Fr}\;B\right), \end{array}$$

for any IFS *B* in *Y*.

**Proof**

Suppose that *f* is intuitionistic fuzzy semi-continuous. Let *B* be an IFS in *Y*.

Then

(52) sFr f−1(B)sCl f−1(B)⋂sCl f−1(B)¯⊆sCl f−1(sCl B)⋂sCl f−1(sCl B−)=f−1(sCl B)⋂f−1(sCl B−)=f−1(sCl B⋂sCl B−)=f−1(sFr B).

Therefore, *s*Fr *f* ^−1^(*B*)⊆*f* ^−1^(*s*Fr *B*).
